# Histological changes secondary to use of anti-angiogenic therapy after interruption of vasa vasorum flow in the descending aorta: results in a porcine model

**DOI:** 10.1590/1677-5449.180095

**Published:** 2019-04-17

**Authors:** Cyro Castro, Adamastor Humberto Pereira

**Affiliations:** 1 Grupo Hospitalar Conceição – GHC, Cirurgia Vascular, Porto Alegre, RS, Brasil.; 2 Universidade Federal do Rio Grande do Sul – UFRGS, Cirurgia Vascular, Porto Alegre, RS, Brasil.

**Keywords:** vasa vasorum, pathologic neovascularization, angiogenesis inhibitors, vasa vasorum, neovascularização patológica, inibidores da angiogênese

## Abstract

**Background:**

Anti-angiogenic regulators may have therapeutic implications for onset and progression of atherosclerosis.

**Objectives:**

To demonstrate histological changes secondary to the use of bevacizumab in the aorta of pigs after interruption of flow in the vasa vasorum.

**Methods:**

Twelve pigs were divided into two groups. The intercostal arteries of the descending aorta were dissected and ligated and wrapped with a polyvinyl chloride membrane. The treatment group received an intravenous dose of bevacizumab. After 15 days, the animals were euthanized and the aorta removed. Histological slides were prepared for control and treatment groups and for non-manipulated areas and analyzed for degree of angiogenesis, injury, inflammation, and intimal thickening. Data were expressed as mean (SD) of scores and groups were compared using the Mann-Whitney test. The Poisson distribution was used to calculate 95% confidence intervals for mean scores, in order to determine effect statistics.

**Results:**

Bevacizumab had adverse effects on all treated pigs. The analysis using a Scale of Magnitudes for Effect Statistics showed a trend toward a decrease in angiogenesis [0.58 (1.79/-0.63)] and injury [0.55 (1.76/-0.66)] and an increase in inflammation [0.67 (1.89/-0.55)] with threshold moderate effects. There was no difference in intimal thickening [0 (1.19/-1.19)].

**Conclusions:**

The medication exhibited a trend toward reduced angiogenesis and injury, but no reduction in the inflammatory process or intimal thickening of the aortic wall. These findings are in disagreement with studies that correlate neovascularization with increased migration of inflammatory cells. Bevacizumab exhibited toxicity in the porcine model.

## Resumo

**Contexto:**

Agentes antiangiogênicos podem ter implicações terapêuticas na progressão e manifestação da aterosclerose.

**Objetivos:**

Demonstrar a alteração histológica secundária ao uso de bevacizumabe na aorta descendente de suínos submetida à interrupção dos *vasa vasorum*.

**Métodos:**

Em doze suínos, divididos em dois grupos, foi realizada dissecção da aorta torácica, além de ligadura das artérias intercostais e proteção com polivinil. O grupo tratamento recebeu dose endovenosa de bevacizumabe. Após 15 dias, os animais foram sacrificados para retirada da artéria e preparo das lâminas histológicas dos grupos tratamento, controle e áreas não manipuladas para análise quanto aos graus de angiogênese, injúria, inflamação e espessamento intimal. A análise estatística foi conduzida através da média e do desvio padrão dos escores. As comparações entre os grupos foram realizadas pelo teste de Mann-Whitney. A distribuição de Poisson calculou os intervalos de confiança de 95% para as médias, a fim de determinar o efeito estatístico.

**Resultados:**

O bevacizumabe causou efeitos adversos em todos os suínos tratados. As variáveis analisadas através da Escala de Magnitude para Efeito Estatístico demonstraram tendência de redução da angiogênese [0,58 (1,79/-0,63)] e da injúria [0,55 (1,76/-0,66)] e aumento da inflamação [0,67 (1,89/-0,55)] no limite do moderado. Não ocorreu diferença no espessamento intimal [0 (1,19/-1,19)].

**Conclusões:**

A medicação utilizada mostrou tendência de redução da angiogênese e da injúria, mas não reduziu o processo inflamatório ou o espessamento intimal da parede arterial. Esses achados contrariam estudos que correlacionam a neovascularização com o aumento da migração de células inflamatórias. O bevacizumabe mostrou toxicidade no modelo suíno.

## INTRODUCTION

The adventitial vasa vasorum (VV) of healthy arteries is responsible for nourishing the outer component of the vessel wall, while the intima is fed by oxygen diffusion from the lumen. Previous studies have shown that arterial wall hypoxia triggers the neoangiogenesis process in an attempt to provide proper nutrition while also triggering migration of inflammatory cells toward the arterial intima.^1^^-^ 3

It has been suggested that the VV and angiogenesis play a key role in atherosclerotic plaque growth and arterial remodeling, and therefore prevention of this process could induce regression of atherosclerotic lesions. The impact of angiogenesis inhibition suggests that suppression of VV expansion may stabilize or reverse disease progression. Anti-angiogenic agents have been shown to reduce neovascular proliferation and plaque development in animal models of atherosclerosis when administered chronically at high doses. Development of angiogenesis regulators could have therapeutic implications for acute onset and progression of atherosclerosis.^4^^,^ 5

The objectives of the present study were to demonstrate changes in histological response to interruption of VV flow in the descending aorta of pigs associated with use of anti-angiogenic therapy, aiming to reduce vascular proliferation in the aortic wall, and to determine whether there is an influence on intimal thickening, degree of inflammation, and degree of injury.

## MATERIALS AND METHODS

An experimental study was conducted with 12 crossbred pigs (Large White × Landrace) aged 60 to 80 days and weighing 20 to 30 kg each. The animals were randomly divided into two groups, treatment or control (n = 6 each). The model of aortic wall injury adopted in this study, combined with evaluation of changes in responses with systemic anti-angiogenic therapy, has no similar examples in the literature reviewed. The sample was defined based on experimental studies in the same line of research.^4^^,^ 6 ^,^ 7

All experimental procedures were conducted in the Animal Experimentation Unit at the University Hospital Experimental Research Center and the study was approved by the Institutional Ethics Committee (protocol no. 11-0515). All animals were housed in an appropriate unit and received the same balanced age-appropriate diet, without lipid supplementation. Animal handling and experimentation complied with international standards, the Guide for the Care and Use of Laboratory Animals, and Brazil’s guidelines on ethical principles of animal experimentation. All efforts were made to minimize pain and discomfort, as well as to use only the minimum number of animals required to acquire reliable scientific data. The study was conducted in two phases.

### Phase 1

Pigs in the treatment group were premedicated with cimetidine (10 mg/kg) and ondansetron (4 mg) for gastric protection. No anti-inflammatory drugs were used for analgesia due to the adverse effects of bevacizumab. All animals received single-dose antibiotic prophylaxis (cephalothin 1 g). Procedures were performed under general anesthesia with endotracheal intubation, mechanical ventilation, and continuous hemodynamic and electrocardiographic monitoring. During the immediate postoperative period, tramadol (2 mg/kg) was administered intramuscularly to pigs in both groups and ketoprofen (2 mg/kg) was administered only to controls.

Before surgery, the incision site was washed, shaved and cleaned with iodine alcohol 2%. A left lateral thoracotomy incision was made in the 6th left intercostal space, with dissection by planes. After circumferential dissection of the peri-aortic fat, the intercostal arteries were ligated along a 5cm segment distal to the emergence of the left subclavian artery and the dissected aortic area was wrapped with polyvinyl chloride (PVC). Pigs in the treatment group received a single intravenous dose of bevacizumab (5 mg/kg), according to the protocol for metastatic colorectal cancer. In both groups, chest drainage was performed with continuous suction until the end of the procedure. The incision was closed by planes with 0 and 3-0 monofilament nylon suture.

### Phase 2

After 15 days, a surgical technique similar to that performed in Phase 1 was conducted, with the addition of dissection of the entire thoracic aorta, allowing excision of the aortic segment from both surgically manipulated and non-surgically manipulated areas. Patency of the thoracic aorta was determined by presence of a palpable arterial pulse distal of the dissected area. The excised segments were irrigated with sodium chloride 0.9%, fixed in 10% formalin and embedded in paraffin for histological analysis.

After specimen collection, the pigs were euthanized under general anesthesia with a lethal intravenous dose of potassium chloride 50% (1mL/kg), which exerts cardiotoxic effects that quickly cause cardiac arrest.

For histological analysis, two paraffin blocks were prepared for each pig, one containing the middle part of the surgically manipulated aorta and the other containing the middle part of the non-surgically manipulated segment of the aorta. From each paraffin block, 4µm-thick sections were cut and mounted on glass slides. All slides were stained with hematoxylin and eosin (HE), Masson's trichrome stain, and Weigert-van Gieson stain for analysis of elastic fibers.

The following variables were analyzed: angiogenesis, injury, inflammation, and intimal thickening. The degrees of angiogenesis, injury and inflammation were analyzed against criteria adapted from the consensus published by Schwartz et al.^8^ which has been used in our unit,^6^ and scored on scales from 0 to 3.

Angiogenesis: (0) absent; (1) involving the outer third of the media; (2) involving one to two-thirds of the media; and (3) involving more than two-thirds of the media.

Injury: (0) preserved media; (1) tissue disruption in the outer third of the media; (2) tissue disruption in the outer two-thirds of the media; and (3) tissue disruption in entire media.

Inflammation: (0) absence of inflammatory cells; (1) mild inflammatory infiltrate in the outer third of the media; (2) moderate inflammatory infiltrate involving one to two-thirds of the media; and (3) dense inflammatory infiltrate in the entire media.

Intimal thickening was graded as absent, minimal, or mild, based on the amount of smooth muscle cells in the intima (adapted from Suzuki et al.).^9^

Data for all variables are expressed as mean (SD). Control and treatment groups were compared using the nonparametric Mann-Whitney test. The Poisson distribution was used to calculate 95% confidence intervals (95%CI) for the mean scores in order to determine effect statistics using a scale of magnitudes.^10^ The level of significance was set at *p* ≤ 0.05. Statistical analysis was performed using SPSS, version 22.0.

## RESULTS

Bevacizumab had adverse effects on all treated pigs, especially hypotension, bleeding, and gastric lesions. One animal in the treatment group was excluded from the study due to death after Phase 1, caused by a perforated gastric ulcer and peritonitis.

The surgically manipulated segment of the aorta wrapped with the PVC membrane was free from adhesions to the surrounding tissues, and wall thickening was macroscopically evident when compared to the non-surgically manipulated segment of the same aorta, where no changes indicative of angiogenesis, injury, inflammation, or intimal thickening were found (scores of 0).

**Figure 1:**
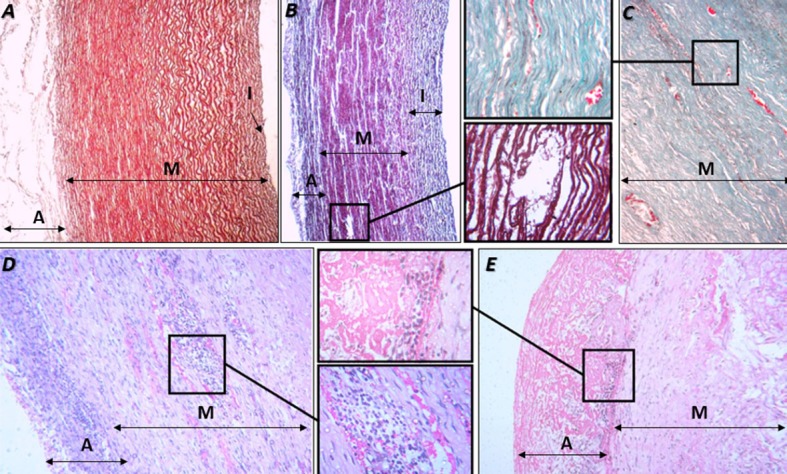
Photomicrograph of the aorta (partial). A) non-surgically manipulated area. Weigert-van Gieson stain (40x). B-E) surgically manipulated area, as follows: B) artery with intimal thickening. Weigert-van Gieson stain (40x). Detail: tissue disruption in the media layer (400x). C) media layer. Masson’s trichrome stain (100x). Detail: vasa vasorum and neoangiogenesis (400x). D) Adventitia and media layers. Hematoxylin and eosin (HE) stain (100x). Detail: inflammatory infiltrate and neoangiogenesis in the media layer (400x). E) Adventitia and media layers. HE stain (100x). Detail: inflammatory infiltrate and disorganization of the adventitia (400x). A = adventitia; I = intima; M = media.

In the photomicrographs, when comparing the non-manipulated area (Figure 1A) with the manipulated areas of the aortic wall, intimal thickening, disruption of elastic fibers, and inflammatory infiltrate were observed in the outer third of the media and adventitia in the control and treatment groups (Figures 1111E).

The histological findings regarding angiogenesis, injury, inflammation, and intimal thickening in surgically manipulated specimens of bevacizumab-treated and control animals are shown in Table 1. There were no significant differences between control and treatment groups in any of the histological parameters analyzed. The results obtained from the Poisson distribution are shown in Table 2.

**Table 1:** Histological analysis of the surgically manipulated parts of the aorta.

	**Control group**		**Treatment group**		***p*-value**
	**Mean**	**SD**	**Mean**	**SD**	
Angiogenesis	2.17	0.41	1.80	0.84	0.338
Injury	2.17	0.41	2.00	0.00	0.361
Inflammation	1.00	0.00	1.20	0.45	0.273
Thickening	2.00	0.00	2.00	0.71	0.99

SD = standard deviation.

**Table 2:** Results of the Poisson distribution.

	**Control group**			**Treatment group**		
	**Mean**	**SD**	**95%CI**	**Mean**	**SD**	**95%CI**
Angiogenesis	2.17	0.15	1.89-2.49	1.80	0.33	1.25-2.59
Injury	2.17	0.15	1.89-2.49	2.00	0.00	2.00-2.00
Inflammation	1.00	0.00	1.00-1.00	1.20	0.18	0.90-1.61
Thickening	2.00	0.23	1.59-2.52	2.00	0.28	1.52-2.64

SD = standard deviation; CI = confidence interval.

**Figure 2:**
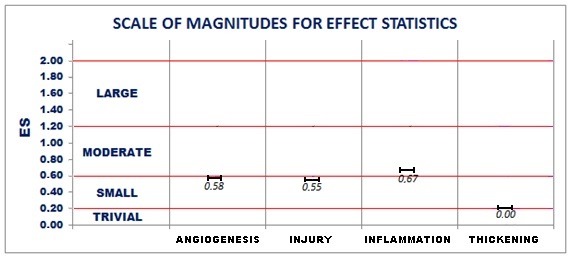
Graphical representation of the scale of magnitudes for effect statistics (ES).

Data obtained from the Poisson distribution were used to calculate effect statistics for each variable, as follows: 0.58 (1.79 to -0.63) for decrease in angiogenesis; 0.55 (1.76 to -0.66) for decrease in injury; 0.67 (1.89 to -0.55) for increase in inflammation; and 0 (1.19 to -1.19) for intimal thickening. The analysis using the Scale of Magnitudes for Effect Statistics^10^ showed a trend toward a decrease in angiogenesis and injury and an increase in inflammation, but there was no effect statistic for intimal thickening (Figure 2).

## DISCUSSION

The similarities between pigs and humans in terms of anatomy, physiology, and pathophysiology have been widely established, particularly with regard to cardiovascular anatomy, vascular response to injury, and clotting and fibrinolytic systems, supporting the choice of pigs as the experimental animal model for this study.^6^^,^ 11 ^,^ 12

By itself, the mechanical injury to the artery induces an angiogenic response and intimal thickening.^13^^,^ 14 Intimal thickening would contribute to arterial wall hypoxia, compromising the supply of nutrients to the inner layers of the tunica media. Previous studies in the aorta of dogs also indicate that interruption of VV flow produces acute changes in distensibility and structural changes in the aortic wall, supporting this concept.^15^^,^ 16 In a study in pigs subjected to resection of the adventitia associated with intercostal artery ligation and coating of the aorta with PVC, Angouras et al.^7^ reported that, after 15 days, interruption of VV flow resulted in ischemic necrosis of the outer layers of the tunica media, with loss of elastin and collagen fiber architecture. In an experimental study in pigs, Fagundes et al.^6^ concluded that removal of the aortic adventitia led to degenerative changes in the media, resulting in loss of the aortic wall structure.

In contrast with other studies described in the literature, this experimental design investigates the effects of arterial wall hypoxia of the thoracic aorta by ligating the intercostal arteries, without removing the adventitia, so that injury is minimized and does not interfere with histological findings or the effects of systemically administered anti-angiogenic therapy. Because peri-aortic tissue may adhere to the dissected area and contribute to neovascularization and migration of inflammatory cells, the aorta was wrapped with a PVC membrane in order to avoid confounding interpretation of the results.

This study showed that, in 15 days, angiogenesis, injury, inflammation, and intimal thickening developed markedly after intercostal artery ligation, even without direct injury to any of the aortic wall layers, leading to early formation of new vessels in the tunica media. Arterial wall hypoxia was sufficient to degrade the elastic fibers in the outer third of the tunica media in all specimens, which is consistent with previous findings.^6^^,^ 15 ^,^ 17 ^,^ 18

Over recent years, an increasing number of anti-angiogenic therapies have been proposed that are intended to interfere with modulation of neovascularization and its consequences for diseases such as cancer and macular degeneration. Knowledge of the mechanisms responsible for vascular adventitial proliferation and their extension into the intima is required for study of anti-angiogenic therapies applicable to atherosclerosis.^5^^,^ 19

Neovascularization is mainly mediated by vascular endothelial growth factor (VEGF), which is a potent regulator of the pathophysiology of angiogenesis. Inhibition of plaque neovascularization is a potential therapeutic target. Bevacizumab, a recombinant monoclonal antibody against VEGF, has been used recently in the form of drug-eluting stents to treat restenosis in coronary arteries; safely and associated with minimal neointimal hyperplasia.^5^^,^ 18

In vivo studies have shown that blocking formation of new vessels can significantly reduce intimal plaque size.^19^ Moulton et al.^20^ demonstrated that two angiogenesis inhibitors, endostatin and TNP-470, reduced formation of new vessels and intimal plaque growth in the aorta of mice. Stefanadis et al.^21^ showed that use of antibodies specific for VEGF resulted in a significant reduction in neovascular growth and neointimal thickening after 4 weeks in rabbits on an atherogenic diet. Gossl et al.^22^ reported that inhibition of VV neovascularization using thalidomide in pigs on a high-cholesterol diet significantly reduced neointimal thickening and plaque development. Mollmark et al.^23^ demonstrated that the anti-angiogenic activity of rPAI-123 (a truncated plasminogen activator inhibitor-1 protein) induces VV collapse and significantly reduces cholesterol and plaque size in hypercholesterolemic mice. Kampshulte et al.^24^ conducted an experimental study in mice fed a western diet with and without thalidomide for 29 weeks, using nano-computed tomography for evaluation of the descending aortas, and showed that the cross-sectional area of the adventitial VV was significantly decreased in mice treated with thalidomide, accompanied by a decrease in the total number of VV per cross-sectional image, demonstrating a significant reduction in atherosclerotic plaque area. However, none of the studies mentioned above tested the action of the drug on arteries subjected to ischemia of the wall by decreased VV flow, as induced in this experiment.

The pathophysiological concepts that the inner layers of the atherosclerotic plaque undergo a process of ischemia, which induces formation of new vessels from the VV, and that this process induces influx of a greater number of inflammatory cells into the arterial wall are all well-defined in the literature.^2^^,^ 4 ^,^ 19 The purpose of this study was to investigate the action of a recombinant monoclonal antibody against VEGF (bevacizumab), at a dosage of 5mg/kg, according to the standard protocol for metastatic colorectal cancer, and test the hypothesis that use of anti-angiogenic therapy would reduce histological changes secondary to hypoxia induced by interruption of VV flow in the descending aorta of pigs, by assessing vascular proliferation and the degree of injury, inflammation, and intimal thickening.

The pigs were very sensitive to the adverse effects of bevacizumab. All animals in the treatment group required special care during the intraoperative and postoperative periods because of the adverse effects of this medication. All six pigs in the treatment group exhibited hypotension during drug administration and increased bleeding, requiring aggressive fluid resuscitation with crystalloids and colloids and, in two cases, intraoperative vasopressor use.

Postoperatively, the first and second pigs in the treatment group had vomiting, diarrhea with melena, hematuria, anorexia, and abdominal pain, and treatment was started with cimetidine, sucralfate, ondansetron, and metoclopramide. Abdominal ultrasound showed free fluid in the cavity. Despite treatment, the first pig died on postoperative day 3 and necropsy revealed a perforated gastric ulcer with peritonitis; this animal was excluded from the study. The second pig progressed favorably. At the time of euthanasia, which was the date scheduled for specimen collection, this pig also had a gastric ulcer at necropsy. A protocol for gastric protection with preoperative and postoperative administration of the above-mentioned drugs was established and use of anti-inflammatory drugs was contraindicated, achieving acceptable control of these postoperative adverse effects in the next four pigs operated on in this group.

Sample size calculation was based on studies of the same topic, but the experimental model used in the present study has unique features and no parallel in the literature; therefore, there are no objective data to serve as a basis for calculation. Statistical significance was not achieved for the variables analyzed. The non-equivalence between statistical significance and clinical importance is well recognized and was used to complement statistical analysis of the data.^25^

Based on the present findings, the magnitude of effect statistics^10^ was determined for each variable. Analysis of the results showed a trend toward a reduction in angiogenesis and the degree of injury to the vessel wall with use of bevacizumab, with a statistical effect close to moderate. Conversely, there was a trend toward worsening of the degree of inflammation with use of this monoclonal antibody, also with a moderate statistical effect, which is in contrast to the findings of some studies that anti-angiogenic therapy would reduce this response and, consequently, delay or reduce the atherosclerotic process.^3^^,^ 13 ^,^ 19

There was no change in intimal thickening in either group, despite the differences reported in other variables, with a statistical effect equal to zero for this variable (Figure 2). These findings raise the possibility that, perhaps, the potential to stabilize or reduce complex plaques induced by anti-angiogenic agents is limited to only the ability to reduce the risk of intraplaque hemorrhage, having no influence on reducing the degree of inflammation or intimal hyperplasia.

## CONCLUSIONS

In this study, systemic bevacizumab use in a porcine model of aortic wall injury, subjected to hypoxia, showed a trend toward reduced angiogenesis and vessel wall injury. However, there was no reduction in the inflammatory process or intimal thickening of the arterial wall with the anti-angiogenic therapy, even when the magnitudes of the effect statistics were determined. On the contrary, there was a trend to enhanced inflammation. This finding is in disagreement with previous studies that have shown that neovascularization was associated with enhanced inflammation of the arterial wall. Finally, bevacizumab exhibited toxicity in this animal model, as evidenced by hemodynamic changes, blood dyscrasia, and severe gastric lesions, with visceral drilling requiring intraoperative and postoperative intensive care. There is still no conclusive definition of the role neovascularization plays as a response to arterial wall hypoxia. Rather, this is an intricate process that requires further study.
